# Enzymatic Properties of the Neuraminidase of Seasonal H1N1 Influenza Viruses Provide Insights for the Emergence of Natural Resistance to Oseltamivir

**DOI:** 10.1371/journal.ppat.1000103

**Published:** 2008-07-25

**Authors:** Marie-Anne Rameix-Welti, Vincent Enouf, Frédérique Cuvelier, Patricia Jeannin, Sylvie van der Werf

**Affiliations:** 1 Unité de Génétique Moléculaire des Virus Respiratoires, URA3015 CNRS, EA302 Université Paris Diderot, Paris, France; 2 National Influenza Center (Northern-France), Institut Pasteur, Paris, France; The Scripps Research Institute, United States of America

Surveillance of the antiviral susceptibility of influenza viruses in Europe revealed the emergence of influenza A(H1N1) viruses naturally resistant to the anti-neuraminidase inhibitor oseltamivir (Tamiflu) [1]. Currently, resistant viruses are most prevalent in Europe (25%) but less prevalent in the Americas (16%) or the Western Pacific region (4%) [2]. In Europe, the prevalence varies between countries, with highest levels in Norway (66.5%) and France (46.6%) [3]. These frequencies are in sharp contrast with those observed for H1N1 viruses during previous seasons (0 to <1%) [4]–[8].

Resistance was linked to the H275Y mutation (H274Y in N2 numbering) of the N1 known to confer high level resistance to oseltamivir but not to the other anti-neuraminidase inhibitor, zanamivir (Relenza) [9]–[12]. Resistant H1N1 viruses with the H275Y change have been isolated from patients treated with oseltamivir and more frequently in children, especially in Japan, the country with the highest per capita usage of oseltamivir [10],[13]. The current frequencies of resistant H1N1 viruses are not correlated with oseltamivir usage, which suggests that selective drug pressure has not been associated with continued transmission, although it may have been involved in their initial emergence. Clinical H1N1 isolates with the H275Y mutation were previously found to be generally less fit in terms of replication, infectivity for mice or ferrets, or transmission potential [14],[15], although the mutation had a less pronounced and variable effect on virus fitness for laboratory strains such as WSN or PR8 viruses or for H5N1 viruses [9], [16]–[18]. To understand the molecular basis of the apparent fitness of the resistant H1N1 viruses that emerged during the 2007–2008, season we determined the enzymatic characteristics of their neuraminidase.

A selection of H1N1 viruses isolated by the National Influenza Center (Northern-France) from specimens received in the frame of routine surveillance through the GROG sentinel network between weeks 35/2007 and 03/2008 (Table 1) were studied. Using a standard neuraminidase inhibition assay, the IC50 values for oseltamivir ranged from 1.3 to 5.9 nM for sensitive viruses and were much higher (IC50, 624 to 942 nM) for resistant viruses (Table 1), as previously published [9],[10],[12],[19]. All viruses were sensitive to zanamivir (IC50, 1.2 to 3.0 nM). All resistant viruses harbored the H275Y substitution in their N1.

**Table 1:** Properties of the Neuraminidase of H1N1 Viruses from the 2007–2008 Season

Virus^a^	Week^b^	IC50^c^ (nM)		Mean K_m_ d (μM)		Mean V_m_ d (U/sec)		HA titer^e^	K_i_ f (nM)				Amino Acid Position (N1 Numbering)								
		OC	Zana	K_m_ g	Mean^h^	V_m_ g	Mean^h^		OC^i^		Zana^i^		275	78	214	222	249	287	329	344	354
										Mean^j^		Mean^j^									
NC99				27.4	28.0±2.7; *p*<0.001	0.9	1.3±0.45; *p*<0.001	64	0.19	0.21±0.02; *p*<0.05	0.21	0.20±0.04; *p*<0.001	H	K	E	R	G	T	K	D	G
0650/04				25.6	—	2.0	—	256	0.20	—	0.20	—	H	K	E	R	G	T	K	D	G
1951/06				29.5	—	0.85	—	90	0.21	—	0.23	—	H	K	E	R	G	T	K	D	G
0692/07				31.5	—	1.42	—	90	0.19	—	0.16	—	H	K	E	R	G	T	K	D	G
SI06				25.1^***^	—	1.35	—	90	0.22	—	0.17	—	H	K	E	R	G	T	K	D	G
0006/07	35	2.7	1.9	12.4^**^±0.6	*p*<0.001	0.63		64	0.15		0.17		H	K	**G**	**Q**	G	T	**E**	N	G
0286/07	44	3.5	2.9	8.5	9.0±1.2	2.6	3.1±0.75	181	0.086	0.13±0.07	0.081	0.08±0.01	H	**E**	**G**	**Q**	**K**	**I**	**E**	N	D
0497/07	48	4.1	2.7	7.5^**^	—	2.43	—	90	0.160	—	0.075	—	H	**E**	**G**	**Q**	**K**	**I**	**E**	N	D
0611/07	49	4.2	3.0	8.0^*^	—	4.3	—	128	0.101	—	0.10	—	H	**E**	E	**Q**	**K**	**I**	**E**	N	D
0814/07	50	1.9	2.0	9.0	—	2.51	—	90	0.077	—	0.083	—	H	**E**	**G**	**Q**	**K**	**I**	**E**	N	D
0974/08	2	1.3	2.3	10.4^*^	—	3.54	—	181	0.079	—	0.086	—	H	**E**	**G**	**Q**	**K**	**I**	**E**	N	D
1149/08	2	5.9	1.4	10.4	—	3.48	—	90	0.249	—	0.08	—	H	**E**	**G**	**Q**	**K**	**I**	**E**	N	D
0341/07	45	732	2.3	18	19.4±2.9; *p*<0.001	2.46	3.4±1.52; NS	90	55	58±11; *p*<0.001	0.18	0.19±0.04; *p*<0.001	**Y**	**E**	**G**	**Q**	**K**	**I**	**E**	N	G
0577/07	49	852	2.7	19.5	—	1.98	—	90	52	—	0.18	—	**Y**	**E**	**G**	**Q**	**K**	**I**	**E**	N	G
0644/07	50	918	3.0	18.4^**^	—	2.95	—	181	58	—	0.22	—	**Y**	**E**	**G**	**Q**	**K**	**I**	**E**	N	G
0749/07	51	720	1.4	18	—	3.43	—	181	47	—	0.10	—	**Y**	**E**	**G**	**Q**	**K**	**I**	**E**	N	G
0847/07	52	624	1.3	24	—	3.04	—	ND	53	—	0.17	—	**Y**	**E**	**G**	**Q**	**K**	**I**	**E**	N	G
0910/08	1	696	1.3	25	—	5.76	—	181	55	—	0.18	—	**Y**	**E**	**G**	**Q**	**K**	**I**	**E**	N	G
0963/08	1	690	1.7	18.4^*^	—	2.75	—	90	51	—	0.24	—	**Y**	**E**	**G**	**Q**	**K**	**I**	**E**	N	D
1154/08	2	642	1.2	14.5	—	4.4	—	181	59	—	0.17	—	**Y**	**E**	**G**	**Q**	**K**	**I**	**E**	N	G
1157/08	3	942	1.4	20	—	1.2	—	90	82	—	0.19	—	**Y**	**E**	E	**Q**	**K**	**I**	**E**	N	G
1170/08	3	708	1.4	18.2^*^	—	6.25	—	181	75	—	0.26	—	**Y**	**E**	**G**	**Q**	**K**	**I**	**E**	N	G
1208/08	3	630	1.9	19	—	3.13	—	90	49	—	0.17	—	**Y**	**E**	**G**	**Q**	**K**	**I**	**E**	N	G

*p-*Values are the result of the Student's t test between the mean of K_m_, V_m_, or K_i_ of the sensitive viruses versus either the resistant ones or the pre-2007–2008 viruses. Student's t test was also performed between the mean K_m_ of the sensitive viruses versus the mean K_m_ of the early seasonal virus #0006/07.

Amino acids that differ from the NC99 strain are shown in bold.

a Except for the reference vaccine strains A/New Caledonia/20/99(H1N1) (NC99) and A/Solomon Islands//2006(H1N1) (SI06), all other viruses were A(H1N1) clinical isolates from the NIC (Northern-France) named by order number and year of isolation. Virus 0692/07 is an isolate from the 2006–2007 season.

b Week of sampling of specimen during the 2007–2008 season.

c IC50 determined essentially as described in [20],[30] using the MUNANA substrate at a final concentration of 100 μM.

d The Michaelis-Menten constant K_m_ was determined by enzymatic kinetic analyses performed on inactivated virus suspensions. Briefly, kinetics were performed using MUNANA concentrations ranging from 5 to 100 μM. Initial velocity of the reaction was calculated and plotted as a function of the MUNANA concentration. K_m_ and V_m_ were calculated using a nonlinear regression of the curve according to the Michaelis-Menten equation. V_m_ values were expressed in arbitrary units (U/sec).

e Hemagglutination (HA) titers were determined in duplicate by standard procedures using guinea pig red blood cells. Titers are expressed as the mean of the reciprocal of the last virus dilutions showing hemagglutination.

f K_i_ determinations rely on enzymatic kinetic analyses. The kinetics were performed in the presence of variable concentrations of inhibitor (0 to 2,000 nM) and a constant MUNANA concentration (20 μM). Calculation of the K_i_ was performed by nonlinear regression of the plot of the initial velocity as a function of the concentration of inhibitor.

g K_m_ and V_m_ values are given as the mean of 2, 3^*^, 4^**^, or 7^***^ determinations.

h Values correspond to the mean ± standard deviation of the mean K_m_ or V_m_ values of the 5 pre-2007–2008 viruses , the 6 sensitive viruses, and the 11 resistant viruses. Means and *p*-values apply to virus groups indicated by —.

i K_i_ values were determined once except for the SI06 reference strain, for which three independent determinations were performed, and the 0497/07 and 644/07 viruses, for which two independent determinations were performed.

j Values correspond to the mean ± standard deviation of the mean K_i_ values of the 5 pre-2007–2008 viruses, the 6 sensitive viruses, and the 11 resistant viruses. Means and *p*-values apply to virus groups indicated by —.

ND, not determined; NS not significant.

Kinetic analyses of sialidase activities of the neuraminidase were performed using the MUNANA fluorogenic substrate in the absence or presence of neuraminidase inhibitors on whole virus suspensions as described [20]. The Michaelis-Menten constant (K_m_), which reflects the affinity for the substrate, and the V_m_, which reflects the activity of the enzyme, were determined (Table 1). The K_m_ values for the MUNANA substrate of most viruses from the 2007–2008 season sensitive to oseltamivir (9.0±1.2 μM) were significantly reduced as compared to those measured for the A/New Caledonia/20/99(H1N1) (NC99) and A/Solomon Islands/3/2006(H1N1) (SI06) vaccine strains and for sensitive H1N1 isolates from previous seasons (28.0±2.7 μM). One virus (#0006/07) showed an intermediate K_m_ (12.4±0.6 μM). The mean K_m_ values for MUNANA (19.4±2.9 μM) were significantly (*p*<0.001) higher for viruses resistant to oseltamivir as compared to sensitive viruses, as previously reported [11],[20]. However, K_m_ values (19.4±2.9 μM) for resistant viruses from the 2007–2008 season remained below the K_m_ values (28.0±2.7 μM; *p*<0.01) for NC99 and SI06 vaccine strains and sensitive H1N1 isolates from previous seasons. Analysis of the V_m_ values showed no significant difference for sensitive as compared to resistant viruses from the 2007–2008 season (3.1±0.75 and 3.4±1.52 U/sec, respectively). However, the N1 of viruses circulating prior to 2007–2008 exhibited significantly lower V_m_ values (1.2±0.47 U/sec; *p*<0.05) than that of viruses from the 2007–2008 season except for isolate #0006/07, which had a low V_m_ value (0.63 U/sec).

Inhibition constants (K_i_) for oseltamivir and zanamivir were also determined (Table 1). As for the K_m_ values, K_i_ values for zanamivir and oseltamivir were significantly and about 2-fold lower for the 2007–2008 viruses sensitive to oseltamivir (except for isolate #0006/07) as compared to NC99 and SI06 vaccine strains and sensitive H1N1 isolates from previous seasons. As expected, for the 2007–2008 viruses resistant to oseltamivir, mean K_i_ values for oseltamivir were more than 500-fold higher than for their sensitive counterparts (58±11 and 0.13±0.07 nM; *p*<0.001), albeit reduced about 2-fold when compared to values previously reported for resistant H1N1 viruses (105 to 200 nM; [11],[15],[20]). Thus, the neuraminidase of H1N1 viruses from the 2007–2008 season exhibits an increased affinity for the substrate as well as for the two anti-neuraminidase inhibitors and a higher activity as compared to previously circulating viruses such as NC99 or SI06, except for isolate #0006/07, which behaved as an intermediate. As a result, the neuraminidase from recent resistant viruses that harbor the H275Y substitution has a slightly higher activity and affinity for the substrate than that from previously circulating sensitive viruses. These features may contribute to their overall fitness and ability to be transmitted, although the contribution from other genes cannot be excluded at present.

When comparing the growth characteristics in vitro on MDCK SIAT-1 cells of the resistant viruses with that of sensitive viruses from the 2007–2008 season or from previous seasons, no significant differences in growth kinetics or final virus titers were observed (Figure 1). These results indicated that, at least in vitro, the presence of the H275Y mutation did not significantly impair the fitness of the viruses, unlike what had been previously reported in the case of the A/Texas/36/91 virus on MDCK cells [15]. Whether the same holds true in vivo remains to be determined.

**Figure 1:**
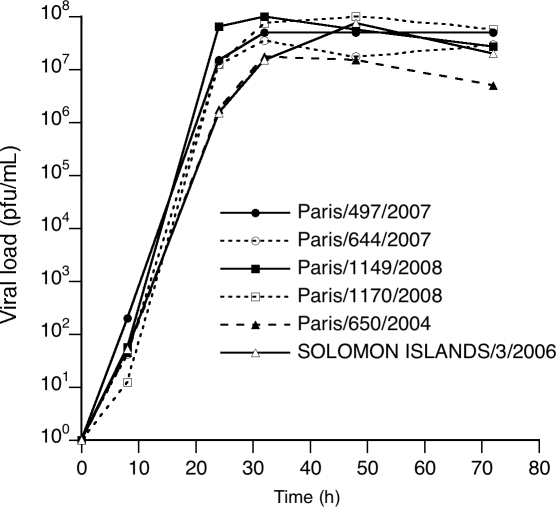
Growth Kinetics of H1N1 Viruses from the 2007–2008 Season Sensitive or Resistant to Oseltamivir. Two sensitive (A/Paris/497/2007 and A/Paris/1149/2008) and two resistant (A/Paris/644/2007 and A/Paris/1170/2008) viruses from the 2007–2008 influenza season, as well as the reference strain A/Solomon Islands/3/2006 and an isolate from the 2003–2004 season (A/Paris/650/2004), were amplified and titrated on MDCK cells. The indicated viruses were then used to infect MDCK SIAT-1 cells [31] at an m.o.i. of 0.001 and incubated for 72 hours at 35°C in the presence of 1 μg/ml TPCK trypsin. At the indicated time points, the supernatants were harvested and virus titers were determined by plaque assays on MDCK cells.

Phylogenetic analysis of the N1 sequences showed that sensitive and resistant viruses from the 2007–2008 season belong to the same clade, including two viruses from Hawaii (A/Hawaii/21/2007 and A/Hawaii/28/2007) with the H275Y change (Figure 1). Strikingly, isolate #0006/07 (A/Paris/6/2007), which behaved as an intermediate, belonged to a different clade. When analyzing the H1 sequences, again resistant and sensitive viruses belonged to the same clade, including the recent vaccine strain A/Brisbane/59/2007 (Figure 2). No specific amino acid changes that could be compensating for the presence of the H275Y substitution in the N1 were found in the H1 of resistant viruses. For instance, sensitive (#0497/07, #1149/08) and resistant (#0644/07 and #1170/08) viruses with the same HA and NA (except for the H275Y and G354D changes) amino acid sequences representing the consensus sequences of the recent H1N1 viruses had similar growth characteristics, similar V_m_ values for their N1, and differed in their K_m_ and K_i_ values based solely on the two changes in the N1.

**Figure 2:**
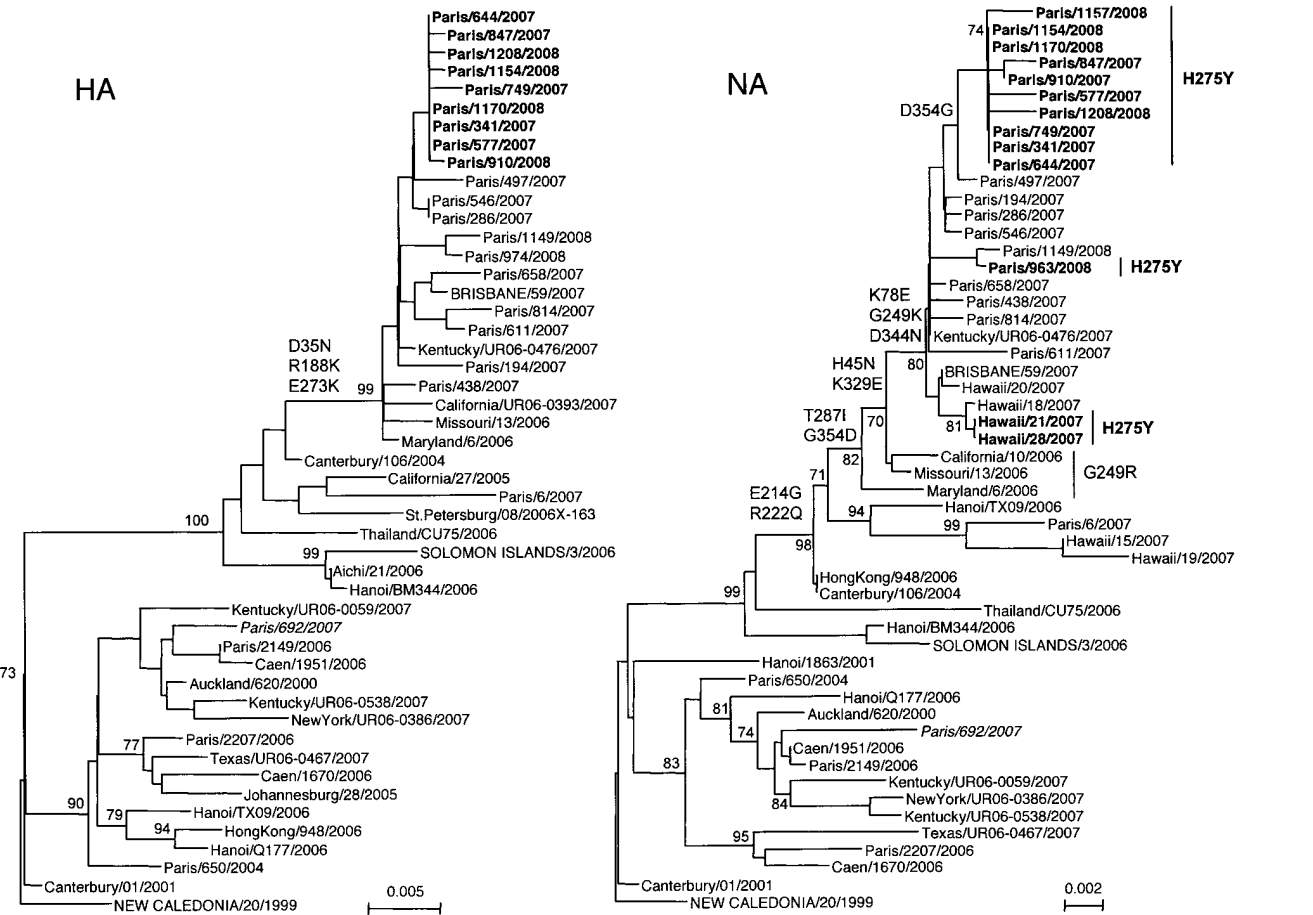
Phylogenetic Relationships of the H1 and N1 Genes. The phylogenetic analysis was performed on the alignment of sequences from nucleotides 87 to 995 (H1) or 90 to 1286 (N1) (numbering from ATG). The dendrogram was constructed by genetic distance matrix and calculated with the DNADIST program using the Kimura-2 parameters model with transition-to-transversion ratio of 2.0 and neighbor-joining analysis in the PHYLIP package [32],[33]. Sequences from the H1 or the N1 from A/NewCaledonia/20/99 were used as outgroup. Bootstrap values of 1,000 replicas are given as percentages at the nodes. Isolates from Northern-France from the 2006–2007 season are in italics. Viruses with a Y275 in the neuraminidase sequence are shown in bold and vaccine strains in capitals. Published sequences were issued from the influenza sequence database at Los Alamos National Laboratory [22].

In addition to the H275Y change, most, but not all, resistant viruses were characterized by the presence of a G354 as for the NC99 and SI06 viruses, whereas a D354 was found for sensitive viruses (Table 1). According to the three-dimensional structure of the N1 of an H5N1 avian influenza virus [21], residue 354 is located on the top external side of the neuraminidase tetramer at a distance from the catalytic site and subunit interfaces. It is therefore not likely to be compensating for the H275Y substitution. Indeed, as shown for isolate #0963/08 as compared to other resistant isolates, the presence of a D354 rather than a G354 does not have a major impact on the enzymatic characteristics of the N1 (Table 1). Substitutions that distinguish the majority of H1N1 viruses from the 2007–2008 season from both NC99 and SI06 are H45N, K78E, E214G, R222Q, G249K, T287I, K329E, and D344N. Two of these positions are located in the stalk region (45 and 78), and three (222, 249, 344) in the vicinity of the catalytic site according to the three-dimensional structure of the N1 [21]. Substitutions in the vicinity of the substrate binding site may influence the affinity of the neuraminidase for its substrate, whereas remote substitutions in the ectodomain are less likely to be significant. Indeed, sensitive (#0611/07) and resistant (#1157/08) viruses with E214 showed similar K_m_ and K_i_ values as their counterparts with G214 (Table 1). According to the N1 sequences available for H1N1 viruses in the ISD database [22], the specific amino acid combination mentioned above emerged in 2007. In particular, a K249 had not been observed previously, and its prevalence increased to reach approximately 85% for 2008 isolates in the database (100% for isolates from Northern-France). Isolate #0006/07, which lacked the G249K change, showed intermediate K_m_ and K_i_ values. Some viruses, such as A/Missouri/13/2006 and A/California/10/2006, were reported to have an R249 in association with the specific combination of amino acids, except for the K78E and D344N changes. It would be of interest to determine their K_m_ and K_i_ values.

Overall, our results suggest that a specific combination of amino acids may have resulted in an increased affinity of the N1 of recent H1N1 viruses for its substrate and neuraminidase inhibitors. It will be of interest to determine more precisely which exact changes are involved through mutagenesis using the previously described transient N1 expression system for kinetic analyses of the neuraminidase activity [20].

Appropriate functional balance between the activities of the two influenza virus glycoproteins towards sialic acids, i.e., receptor binding (hemagglutinin) and sialidase activity (neuraminidase), is essential for virus fitness [23]. The H1 of viruses from the 2007–2008 season differ from both NC99 and SI06 by three substitutions (D35N, R188K, E273K), none of which are involved in direct interactions with the receptor and therefore not likely to result in changes of affinity of the H1 for the receptor. According to this hypothesis, which warrants further experiments, the increased affinity of the N1 of 2007–2008 viruses for its substrate would not have been compensated by an increased affinity of the H1 for the receptor. Therefore, viruses with a Y275 that have only a slightly higher affinity for the substrate as compared to H1N1 viruses that circulated previously may have a more appropriate balance of their hemagglutinin and neuraminidase activities than viruses with a H275 that have a 3-fold increased affinity of their neuraminidase for the substrate. As a result, as for influenza A viruses resistant to adamantanes [24]–[28], the recent resistant viruses would not be outcompeted upon circulation in the community. It should be emphasized, however, that the relative fitness and ability to be transmitted of the resistant versus sensitive viruses may be modulated by characteristics of other genes. This will require whole genome sequencing. The circulation of H1N1 viruses naturally resistant to oseltamivir underlines the fact that genetic variations may result in variations in sensitivity to oseltamivir in the absence of selective drug pressure, as shown for H5N1 viruses [20],[29]. Genetic variations of the hemagglutinin and neuraminidase are mainly driven by the immune response, and adventitious properties that result in changes in fitness may be co-selected. Such a phenomenon could potentially take place for H5N1 viruses and also for H3N2 viruses. Genetic variations like these emphasize the need to carefully monitor the affinity of the neuraminidase for its substrate and anti-neuraminidase inhibitors in relation with the binding affinity of the hemagglutinin for its receptor for influenza viruses circulating in the population, as well as for avian influenza viruses with pandemic potential.

## Sequence Accession Numbers

GenBank accession numbers are listed in Table 2 for the viruses included in this report.

**Table 2:** Accession Numbers

Virus Isolate	H1	N1
A/Paris/0650/2004	EU685784	EU718491
A/Caen/1670/2006	EU551852	EU551817
A/Caen/1951/2006	EU551843	EU551819
A/Paris/2149/2006	EU551844	EU551829
A/Paris/2207/2006	EU551853	EU551816
A/Paris/0692/2007	EU551850	EU551827
A/Paris/0006/2007	EU551851	EU551807
A/Paris/0194/2007	EU551848	EU551808
A/Paris/0286/2007	EU551838	EU551828
A/Paris/0341/2007	EU551832	EU551811
A/Paris/0438/2007	EU551849	EU551830
A/Paris/0497/2007	EU551839	EU551818
A/Paris/0546/2007	EU551837	EU551822
A/Paris/0577/2007	EU551835	EU551815
A/Paris/0611/2007	EU551847	EU551823
A/Paris/0644/2007	EU551833	EU551809
A/Paris/0658/2007	EU551845	EU551820
A/Paris/0749/2007	EU551831	EU551826
A/Paris/0814/2007	EU551840	EU551825
A/Paris/0847/2007	EU551834	EU551824
A/Paris/0910/2008	EU551836	EU551810
A/Paris/0963/2008	ND	EU551821
A/Paris/0974/2008	EU551841	ND
A/Paris/1149/2008	EU685786	EU685787
A/Paris/1154/2008	EU551842	EU551812
A/Paris/1157/2008	ND	EU551814
A/Paris/1170/2008	EU685785	EU685788
A/Paris/1208/2008	EU551846	EU551813

ND, not determined.
